# Immune-Regulatory Mechanisms in Systemic Autoimmune and Rheumatic Diseases

**DOI:** 10.1155/2012/957151

**Published:** 2012-03-07

**Authors:** Britt Nakken, Philip Alex, Ludvig Munthe, Zoltan Szekanecz, Peter Szodoray

**Affiliations:** ^1^Institute of Immunology, Rikshospitalet, Oslo University Hospital, University of Oslo, Oslo 0027, Norway; ^2^Department of Medicine, Johns Hopkins University, Baltimore, MD 21205, USA; ^3^Department of Rheumatology, University of Debrecen Medical and Health Sciences Center, Debrecen 4012, Hungary

Autoimmune diseases encompass a wide range of organ-specific and systemic disorders with a complex etiology. An intricate interplay of genetic, environmental, as well as immunological factors leads to the development of these debilitating diseases. In the absence of infections, regulatory processes inhibit immune responses towards antigen. Moreover, the immune system has multiple levels of negative feedback mechanisms that dampen immune responses and counteract establishment of chronic and destructive immunity. These immune-regulatory functions include a broad spectrum of cellular and molecular mechanisms, which control autoimmune responses. In autoimmune animal models and in patients with autoimmune conditions, various disorders of such regulatory mechanisms have been described. Knowledge and understanding of the immunomodulatory and pathogenic mechanisms that contribute to these conditions can lead to the development of novel diagnostic strategies, and future therapies, providing better life expectancies to patients with autoimmune diseases.

In this special issue, we present original research articles, as well as review papers on the role of derailed regulatory mechanisms underlying autoimmune diseases. 

The paper by Y. Takakubo and Y. T. Konttinen gives an overview of the most important immune-regulatory mechanisms in systemic autoimmune and rheumatic diseases, encompassing the failure of crucial tolerogenic mechanisms, with a special emphasis on tolerogenic dendritic cells, regulatory T and B cells, Th17 cells, inflammatory and tolerogenic cytokines, and intracellular signaling pathways. The paper also introduces the next-generation therapies, beyond the currently used biologic therapies, targeting derailed immune-regulatory processes. 

The paper by C. López-Pedrera et al. addresses epigenetic mechanisms of immune-regulatory functions in conjunction with cardiovascular risk in systemic autoimmune diseases. Epigenetic regulatory mechanisms comprise DNA methylation, histone modifications, and microRNA activity, which influence the development of autoimmune diseases. Other two review articles describe novel immunopathologic roles of diverse cytokines, chemokines, signaling molecules and pattern-recognition receptors in systemic lupus erythematosus, as well as addressing the interaction of CD154 with its various receptors, outlining the role of CD54 in the pathogenesis of lupus and rheumatoid arthritis (RA). 

Three papers present various immune-regulatory mechanisms in connection with RA. 

The paper by J. Furuzawa-Carballeda et al. evaluates the effect of intramuscular administration of polymerized collagen in early and established collagen-induced arthritis (CIA) in mice and analyzes changes in Th subsets following therapy. Polymerized-type I collagen induces upregulation of Foxp3-expressing CD4+ regulatory T-cells and downregulates IL-17-producing CD4+ T-cells (Th17) cells in CIA. Based on these findings, polymerized-collagen may be an effective therapeutic agent in early and established RA by exerting down-regulation of autoimmune inflammation.

 The paper by Y. Shi et al. shows that enhanced high mobility group box chromosomal protein 1 (HMGB1) expression can contribute to Th17 cell activation, and thereby to the perpetuation of autoimmune processes in RA. Another research article in the RA-section of the special issue suggests the Notch pathway may be involved in the pathophysiology of RA, by mediating TNF-*α*-induced IL-6 production in cultured fibroblast-like synoviocytes. 

The paper by B. Szalay et al. assesses the phenotype of T-cell subsets and describes early T-cell activation characteristics in patients with Ankylosing Spondylitis (AS) in conjunction to intravenous therapy with the anti-TNF agent, infliximab. The paper describes that the frequency of Th2 and Th17 cells is higher in AS compared to healthy individuals. This abnormal immune phenotype together with functional disturbances of CD4+ and CD8+ cells in AS can partially be restored by infliximab administration. 

The paper by D. Mieliauskaite et al. describes the expression of IL-17, IL-23 and their receptors in minor salivary glands of patients with primary Sjögren's syndrome.

 The paper by E. D. Abston et al. investigates the role of virus-activated Toll-like receptor (TLR)3 and its adaptor TRIF on the development of autoimmune coxsackievirus B3 (CVB3) myocarditis in mice and shows that TLR3 versus TRIF deficiency results in altered Th2 responses that uniquely influence the progression to chronic myocarditis. 

The paper by B. De Paepe et al. gives an overview on the TNF superfamily of cytokines in idiopathic inflammatory myopathy. For each TNF family member, the possibilities for treating inflammatory diseases in general and the idiopathic inflammatory myopathies in particular are explored.

 The paper by S.-J. Chen et al. introduces the current status of immune-regulatory processes and immunomediated therapeutic strategies for multiple sclerosis and highlights the growing evidence that Th17 cells play a pivotal role in the complex adaptive autoimmunity of the disease and discusses the roles of the associated immune cells and cytokines. 

The paper by N. Rieber et al. presents current concepts of hyperinflammation in the pathogenesis of chronic granulomatous disease (CGD). The paper summarizes the role of reduced neutrophil apoptosis and efferocytosis, dysbalanced innate immune receptors, altered T-cell surface redox levels, induction of Th17 cells, the enzyme indolamine-2,3-dioxygenase (IDO), impaired Nrf2 activity and inflammasome activation, as well as their potential therapeutic implications in CGD. 

This special issue encompasses basic, molecular mechanisms of immune-regulation in connection with autoimmune processes, cellular and molecular immune-regulatory functions, which can aid as biomarkers for diagnostics, as well as potential targeting of the immune-regulatory machinery as part of future therapeutic interventions in patients with autoimmune diseases. 

*Britt Nakken* *Philip Alex* *Ludvig Munthe* *Zoltan Szekanecz* *Peter Szodoray*

